# Multicenter cohort of necrotizing enterocolitis in Latin America: evaluation of risk factors and clinical outcomes in Chilean Hospitals

**DOI:** 10.3389/fped.2026.1877057

**Published:** 2026-10-14

**Authors:** Alejandra Sandoval C., Luis Delpiano M., Miguel Ángel Pantoja H., Marta Aravena U., Jeanne Smith R., Hector Pérez A., Sebastian Espinoza E.

**Affiliations:** 1 Hospital Carlos Van Buren, Valparaíso, Chile; 2 Departamento de Pediatría, Universidad de Valparaíso, Valparaíso, Chile; 3 Hospital Clínico San Borja—Arriarán, Santiago, Chile; 4 Facultad de Medicina, Universidad de Chile, Santiago, Chile; 5 Servicio de Neonatología, Clínica Alemana, Santiago, Chile; 6 Hospital Sótero del Río, Santiago, Chile; 7 Servicio de Neonatología, Hospital Sótero del Río, Santiago, Chile; 8 Instituto de Tecnología Para la Innovación en Salud y Bienestar (ITiSB)—UNAB, Viña del Mar, Chile

**Keywords:** mortality, necrotizing enterocolitis, neonatal intensive care, pneumatosis intestinalis, preterm infant

## Abstract

**Background:**

Necrotizing enterocolitis (NEC) remains a leading cause of neonatal morbidity. While risk factors are well documented in high-income countries, data from Latin American cohorts remain limited.

**Objective:**

We aimed to identify local predictors and clinical outcomes associated with NEC in two Chilean NICUs.

**Methods:**

A prospective multicenter cohort study (2017–2018) included newborns ≤32 weeks and/or ≤1,500 g. NEC (Bell stage ≥ IIA) was verified by a multidisciplinary team. Clinical and laboratory variables were analyzed using bivariate and multivariable penalized models to identify predictors of NEC and mortality.

**Results:**

Among 177 newborns (mean GA 29.1 ± 3.0 weeks; BW 1,252 ± 448 g), NEC incidence was 7.9% (*n* = 14). In bivariate analysis, invasive mechanical ventilation (RR = 4.58; *p* = 0.02) and indomethacin (RR = 3.13; *p* = 0.04) were associated with NEC. In multivariable models, formula feeding was the most consistent predictor (OR = 2.69 vs. exclusive breast milk), though not statistically significant. Half of NEC cases (50%) were Bell stage IIIB. Surgery was required in 38.5% of cases (all stage IIIB; *p* = 0.009). Case fatality among NEC patients was 40%, associated with Bell stage IIIB (*p* = 0.048) and systemic shock (*p* = 0.048).

**Conclusion:**

Formula feeding was a consistent, though non-significant, predictor of NEC in multivariable analysis; the limited number of NEC cases (*n* = 14) precludes definitive causal inference. Exclusive breastfeeding remained the most protective feeding strategy, consistent with prior literature. Disease outcomes were driven by progression to Bell stage IIIB, and early monitoring of inflammatory and metabolic biomarkers may help identify infants at risk of progression to surgical NEC.

## Introduction

Necrotizing enterocolitis (NEC) is one of the most catastrophic gastrointestinal diseases affecting preterm infants, being the leading cause of gastrointestinal illness and mortality in this population (1). Despite advances in neonatal care for smaller preterm infants, NEC remains a high-impact pathology in neonatal intensive care units (NICUs), accounting for 1 to 5% of NICU admissions. Its incidence is higher with lower gestational age and lower birth weight, reaching approximately 22% in extremely low birth weight preterm infants (2–4).

The total mortality rate ranges from 20%–30%, reaching up to 50% in surgical NEC (5). Short-term morbidity arises mainly from surgical complications such as short bowel syndrome, intestinal obstruction, and wound dehiscence, with long-term sequelae affecting pulmonary, gastrointestinal, and neurodevelopmental function (5).

Several risk factors for NEC have been identified. Beyond gestational age, birth weight, and feeding type — the classically recognized determinants (6) — clinical exposures such as invasive mechanical ventilation, red blood cell transfusion, and indomethacin use have been associated with increased risk (7). Recent evidence also suggests that laboratory markers, including C-reactive protein elevation and early thrombocytopenia, may predict progression to surgical NEC at the time of diagnosis (8). The presence of modifiable risk factors is strongly influenced by clinical practice; consequently, standardizing preventive strategies adapted to each local context may reduce NEC-associated morbidity and mortality (9).

Despite this body of evidence, data on NEC epidemiology and risk factors from Latin American NICUs remain scarce (10). Given this knowledge gap, our objective was to characterize the incidence, risk factors, and clinical outcomes of NEC in two Chilean NICUs.

## Methods

Ethics Statement: Information was collected in an anonymized Excel database for subsequent statistical analysis. The respective Ethics Committees approved this study and the corresponding informed consent, number 02032017.

This prospective cohort study enrolled all newborns with gestational age ≤32 weeks and/or birth weight ≤1,500 g, admitted between January 2017 and December 2018. Two public hospitals in Chile participated: Hospital Clínico San Borja Arriarán and Hospital Sótero del Río. Data were collected prospectively in real time using standardized case report forms; written informed consent was obtained from parents or legal guardians prior to enrollment. NEC was defined using the modified Bell staging criteria (11), and cases that met the criteria for classification into stage IIA, IIB, IIIA, and IIIB at the time of clinical presentation were included. The diagnosis was established by the attending neonatologist, and subsequently, all cases were verified by a secondary review of medical records by another unit neonatologist and/or an experienced pediatric infectious disease specialist in neonatology.

The date of NEC diagnosis was defined as the day on which the initial blood culture was obtained and treatment was initiated. Infants were followed clinically until hospital discharge or death, whichever occurred first. The in-hospital mortality outcome was available for 109 of the 177 infants (61.6%) Initial data were collected on paper forms and were not immediately transferred to the electronic database. Because datasets were anonymized, retroactive linkage to original clinical records was not possible. All mortality analyses are therefore restricted to that subsample, and this is stated wherever mortality results are reported.

Patients who presented the following conditions were excluded from the analysis: congenital disease incompatible with life, patients transferred to another hospital, patients born in another hospital, patients transferred from another hospital with a diagnosis of NEC, and patients diagnosed with spontaneous intestinal perforation.

The following risk factors, identified from the literature, were evaluated: perinatal asphyxia (based on cord gas values), type of delivery (vaginal; cesarean); type of feeding (exclusive breast milk, mixed, exclusive formula); prenatal history (intrauterine growth restriction, gestational diabetes, preeclampsia, cerclage, premature rupture of membranes, intra-amniotic infection, maternal antibiotic use, altered maternal and/or fetal Doppler, antenatal corticosteroid); newborn history [number of red blood cell transfusions, polycythemia, invasive mechanical ventilation, vasoactive drugs, days of umbilical catheter (venous, arterial), and antibiotic exposure prior to NEC diagnosis].

Antibiotic exposure was defined as any systemic antibiotic administered to the neonate from birth up to and including the day of NEC diagnosis, regardless of indication. Invasive mechanical ventilation (IMV) was defined as any requirement for endotracheal intubation and ventilation during hospitalization or at the time of NEC diagnosis; IMV initiated as a direct consequence of NEC severity was excluded from this variable to avoid reverse causation. Red blood cell transfusion was operationalized as a binary variable (any transfusion administered from birth to the day of NEC diagnosis vs. none); data on temporal proximity to NEC onset (e.g., within 48–72 h of diagnosis) were not available in this dataset. For infants who did not develop NEC, and who therefore have no diagnosis date on which to anchor these windows, exposures were were determined during the period in which they were hospitalized. The observation periods were consequently not identical in the two groups.

Laboratory variables included: white blood cell count (×10⁹/L), percentage of immature leukocytes, platelet count (×10⁹/L), C-reactive protein (mg/L), and venous blood gases (pH, bicarbonate, and base excess). All laboratory values were recorded at the time of NEC diagnosis. None of the centers used probiotics in their neonatal units.

Feeding protocols during the study period (2017–2018) were not standardized across participating units; feeding decisions were at the discretion of the attending neonatologist. Consequently, detailed data on the timing of enteral feed initiation, rate of volume advancement, duration of nil-per-os periods, use of donor milk, and fortification practices were not systematically collected and could not be included in the analysis.

### Statistical analysis

Continuous variables were summarized using the mean and standard deviation (SD) or the median and interquartile range (IQR), depending on their distribution. Categorical variables were described as frequencies and percentages.

The association between necrotizing enterocolitis (NEC) and clinical covariates was initially explored using contingency tables. When expected cell counts were small or events were infrequent, exact tests were applied and relative risks (RR) with 95% confidence intervals (95% CI) were estimated. The risk of in-hospital mortality associated with NEC was estimated using crude RR in the subsample with observed mortality outcomes, given the substantial proportion of missing mortality data. To further evaluate the plausibility of this pattern, baseline characteristics were compared between infants with and without observed mortality outcomes: missingness was unrelated to NEC status (*p* = 0.43), gestational age, or birth weight, but was significantly associated with invasive mechanical ventilation (*p* = 0.005) and red blood cell transfusion (*p* < 0.001), indicating that mortality data were not missing completely at random.

We specified candidate predictors *a priori* based on risk factors previously reported in the NEC literature. We did not apply an automated or data-driven selection procedure, such as stepwise selection, and we did not retain or remove variables based on their statistical significance in this dataset. Because several specifications and exposures were examined without adjustment for multiple comparisons, the multivariable analyses are reported as exploratory and hypothesis-generating throughout

Because NEC was infrequent and conventional logistic regression models were affected by separation or quasi-separation, we performed multivariable analyses in the full cohort using Firth’s penalized logistic regression and reported odds ratios (OR) and 95% confidence intervals. Models were specified sequentially as follows: (i) a baseline model including perinatal variables, (ii) an expanded clinical model, and (iii) a parsimonious core model excluding pharmacological exposures. The incremental contribution of individual pharmacological variables was subsequently assessed by adding each exposure separately to the core model and evaluating its contribution using Wald tests. Given the limited number of NEC events (*n* = 14), the parsimonious core model comprised approximately eight predictor degrees of freedom, corresponding to an events-per-variable ratio of approximately 1.8 — below the conventional threshold of ten recommended for stable maximum-likelihood estimation. Firth's penalized likelihood approach was adopted specifically to mitigate small-sample bias under this constraint and estimates from the expanded models should accordingly be interpreted as exploratory.

We assessed collinearity between gestational age and birth weight using variance inflation factors, which remained below the conventional threshold of ten (gestational age 4.99; birth weight 5.88); we therefore retained both variables. As a sensitivity check, we refitted the models, entering each variable separately.

Missing covariate data were handled by complete-case analysis: each model was fitted on the observations with complete information for every variable included in that specification, and no imputation was performed. The number of observations entering each model is reported with the corresponding estimates

A secondary analysis restricted to NEC cases was conducted to investigate determinants of disease severity and clinical outcomes within this subgroup. Within this subgroup, associations between NEC severity, surgical intervention, and in-hospital mortality were evaluated using contingency tables and Fisher's exact test, given the small sample size and sparse cell counts. Exploratory multivariable analyses in this subsample were also conducted using Firth’s penalized logistic regression. With mortality data available for ten NEC cases and four observed deaths, these models are reported as exploratory and presented to describe the subgroup rather than estimate effect sizes.

All analyses were performed using Stata version 18.0 (StataCorp, College Station, TX).

## Results

### Baseline characteristics of the neonatal cohort

Of the 182 patients initially enrolled, 5 were subsequently excluded due to transfer to another facility (*n* = 1), delivery at an external institution (*n* = 1), confirmed diagnosis of intestinal perforation (*n* = 2), or insufficient data regarding the development of necrotizing enterocolitis (NEC) (*n* = 1).

A total of 177 newborns were included. Mean gestational age was 29.1 ± 3.0 weeks and mean birth weight was 1,252 ± 448 g; median values were 30 weeks (IQR 4) and 1,300 g (IQR 729), respectively. Sex distribution was balanced, with 51.4% of the cohort being male (Table 1). The overall incidence of necrotizing enterocolitis (NEC) was 7.9% (*n* = 14).

**Table 1:** Baseline demographic and clinical characteristics.

Variable	Value
Sample size	177 newborns
Gestational age (weeks), mea*n* ± SD	29.1 ± 3.0
Gestational age, median (IQR)	30 (4)
Birth weight (g), mean ± SD	1,252 ± 448
Birth weight, median (IQR)	1,300 (729)
Male sex	51.4%
Cesarean delivery	72.6%
Invasive mechanical ventilation	56.7%
Red blood cell transfusion	55.2%
NEC incidence	7.9% (*n* = 14)

### NEC was associated with markers of greater clinical severity in bivariate analysis

In the bivariate analysis, invasive mechanical ventilation (IMV) was significantly associated with a higher risk of NEC (RR = 4.58; 95% CI 1.06–19.83; exact *p* = 0.0249; Table 2 and Figure 1A).

**Table 2:** Bivariate association with NEC (exact risk ratios).

Exposure	RR	95% CI	*p* value
Invasive mechanical ventilation	4.58	1.06–19.83	0.0249
Indomethacin	3.13	1.17–8.37	0.0433
Red blood cell transfusion	2.98	0.86–10.25	0.0906
Antenatal corticosteroids	0.47	0.16–1.40	0.24

**Figure 1:**
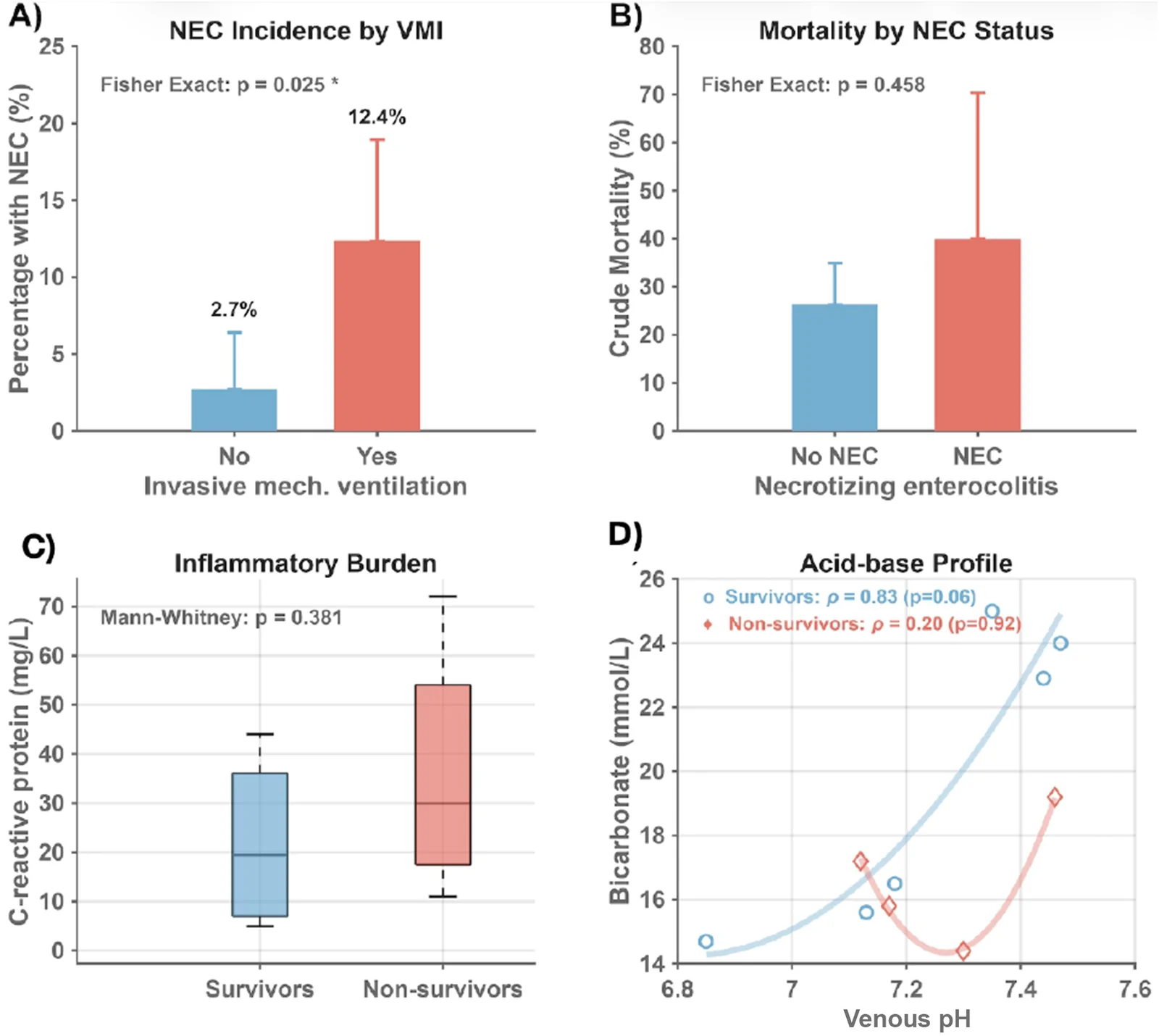
Risk factor analysis, clinical outcomes, and inflammatory markers in newborns with necrotizing enterocolitis (NEC). **(A)** Crude percentage of NEC by invasive mechanical ventilation: Bars represent the proportion of newborns with NEC in each group, and error bars indicate 95% CIs calculated using a binomial approximation. This panel summarizes the unadjusted association between invasive mechanical ventilation and NEC. **(B)** Crude in-hospital mortality by NEC status among newborns with observed mortality outcomes: Bars represent the percentage of deaths within each group, and error bars indicate 95% CIs. Only observations with available mortality data were included in this analysis. **(C)** Inflammatory burden by mortality status among NEC cases: Distribution of C-reactive protein (CRP, mg/L) levels among newborns with NEC, stratified by in-hospital mortality status. Boxes represent the median and interquartile range (IQR), with whiskers indicating the range of observed values. Only NEC cases with available CRP and mortality data were included to provide a descriptive comparison of inflammatory burden between survivors and non-survivors. **(D)** Acid-base profile by mortality status among NEC cases: Scatter plot illustrating the relationship between arterial pH and bicarbonate concentration among newborns with NEC, stratified by in-hospital mortality status. Points represent individual NEC cases, with separate trend lines provided for survivors (red) and non-survivors (grey) to aid visual interpretation. This exploratory figure summarizes acid–base disturbances associated with adverse outcomes and should be interpreted descriptively given the limited sample size.

Consistently, exposure to indomethacin was also significantly associated with NEC (RR = 3.13; 95% CI 1.17–8.37; exact *p* = 0.0433; Table 2). Red blood cell transfusion showed a trend toward increased risk (RR = 2.98; 95% CI 0.86–10.25; *p* = 0.0906), whereas antenatal corticosteroid exposure suggested a potential protective effect, although this did not reach statistical significance (RR = 0.47; 95% CI 0.16–1.40; *p* = 0.24). The use of antibiotics was not a significant risk factor for NEC.

Together, these findings identified variables reflecting greater clinical complexity as relevant risk signals for NEC in the unadjusted analysis.

### NEC was not associated with increased in-hospital mortality in this cohort

Mortality outcomes were available for 109 of the 177 infants (61.6%), and for ten of the fourteen NEC cases, the presence of NEC was associated with a crude relative risk of death of 1.52 (95% CI 0.67–3.49), with no statistically significant difference between groups (*p* = 0.46; Figure 1B).

This pattern was consistent across crude and adjusted analyses; however, no statistically significant association was observed between NEC and in-hospital mortality in this cohort, which may reflect limited statistical power given the small number of NEC cases and the substantial proportion of missing mortality data.

### Feeding type was not associated with NEC in multivariable models

In multivariable penalized models including gestational age, birth weight and feeding type, no feeding category was independently associated with NEC.

In the nutrition-focused Firth's penalized logistic regression model, using exclusive breast milk feeding as the reference category, formula feeding was associated with an increased odds of NEC (adjusted OR = 2.69; 95% CI 0.10–74.46; *p* = 0.56), while mixed feeding also showed higher odds (adjusted OR = 1.38; 95% CI 0.37–5.17; *p* = 0.63), although with wide confidence intervals and without reaching statistical significance.

The confidence intervals around these estimates are too wide to support any conclusion regarding feeding type and NEC risk in this cohort.

### Clinical outcomes and severity among newborns with NEC

Among the 14 newborns diagnosed with necrotizing enterocolitis, disease severity according to the modified Bell classification was distributed as follows: 42.9% were classified as stage IIA (*n* = 6), 7.1% as stage IIB (*n* = 1), and 50.0% as stage IIIB (*n* = 7) (Table 3).

**Table 3:** Disease severity and clinical outcomes among newborns with NEC.

Variable	Value
Sample size (NEC cases)	14
Modified Bell stage	
Bell IIA	6 (42.9%)
Bell IIB	1 (7.1%)
Bell IIIB	7 (50.0%)
Surgical intervention (n)*	5/13 (38.5%)
In-hospital mortality^a^	4/10 (40.0%)
Shock	7/14 (50.0%)
Invasive mechanical ventilation (VMI)	12/14 (85.7%)
C-reactive protein (PCR, mg/L), median (IQR)	10.0 (2.0–34.5)
pH, median (IQR)	7.24 (7.14–7.42)
Bicarbonate, median (IQR)	18.2 (15.7–23.7)
Base excess (EB), median (IQR)	−7.35 (−9.75 to −2.43)

*One observation with missing surgical data.

a Mortality data were available for 10 of the 14 NEC cases.

Surgical intervention was required in 5 of 13 newborns with available data (38.5%). All surgical cases occurred among infants classified as Bell stage IIIB, whereas no surgical interventions were recorded in stages IIA or IIB. This association between disease severity and surgical management was statistically significant (Fisher's exact test, *p* = 0.009).

Mortality data were available for 10 of the 14 NEC cases. Overall, in-hospital mortality among NEC cases with observed outcomes was 40.0% (4/10). All recorded deaths occurred in newborns with Bell stage IIIB NEC, while no deaths were observed among those classified as Bell stage IIA. The single Bell stage IIB case had missing mortality data and was therefore excluded from proportion estimates. Accordingly, a significant association between Bell stage and mortality was observed (Fisher’s exact test, *p* = 0.048).

Exploratory penalized logistic regression analyses using Firth's correction supported these findings. However, when Bell stage and surgical intervention were included simultaneously in mortality models, the association with Bell stage was attenuated, reflecting the strong collinearity between disease severity and the need for surgery.

The presence of shock was significantly associated with in-hospital mortality among NEC cases (Fisher's exact test, *p* = 0.048).

Exploratory analyses of inflammatory and metabolic markers suggested higher C-reactive protein levels and more pronounced acid–base disturbances among non-survivors compared with survivors (Figures 1C,D). Given the small sample size and missing laboratory data, these findings should be interpreted as descriptive and hypothesis-generating.

## Discussion

In our study, the incidence of NEC among those ≤32 weeks and/or ≤1,500 g was 7.9%, a figure consistent with published reports, which estimate a global incidence of approximately 6%–7% (10). Regional differences in NEC incidence between low- and high-income settings have been reported; however, the scarcity of data from low-income countries may limit a full understanding of these differences (10).

Antenatal corticosteroid exposure was the most consistently identified prenatal protective factor in this cohort, consistent with findings from multiple prior studies (12–15).

Although indomethacin exposure and invasive mechanical ventilation were associated with NEC in the bivariate analysis, neither variable retained independent statistical significance when incorporated individually into multivariable penalized models within the parsimonious core framework. This association is widely documented in older studies, but it is not consistently observed, and recent meta-analyses and systematic reviews have dismissed its potential role in favoring NEC (16). The attenuation of these associations after adjustment is consistent with confounding by indication, whereby IMV and indomethacin exposure may act as markers of greater underlying illness severity rather than independent causal contributors to NEC. Beyond confounding by indication, reverse causation is a genuine alternative explanation for several of the exposures examined here. Invasive mechanical ventilation, antibiotic administration, and red blood cell transfusion are all clinical responses to deterioration, and the early, non-specific phase of NEC may itself prompt them. Our operational definition of invasive mechanical ventilation excluded ventilation initiated as a consequence of NEC severity, which limits this concern for that variable, but no equivalent safeguard was available for antibiotic exposure, transfusion or feeding type. These associations should therefore be read as observed within a cohort and not as causes of NEC

Red blood cell transfusion showed a non-significant trend toward increased NEC risk in bivariate analysis (RR = 2.98; 95% CI 0.86–10.25; *p* = 0.09). Transfusion-associated NEC (TANEC) is a recognized clinical entity, with meta-analytic evidence linking transfusion exposure to a substantial increase in NEC risk among very low birth weight infants (17, 18). Both the transfusion event and preceding anemia have been proposed as independent contributors: anemia may compromise intestinal oxygen delivery and mucosal integrity, predisposing the gut to injury when circulatory stress is induced by the transfusion itself, and the combined effect appears additive (19–21). Feeding management during the peritransfusional period may further modulate this risk, and future prospective studies in these units should record feeding status relative to transfusion timing (22).

We could not demonstrate an association between feeding type and NEC in this cohort. The wider literature strongly supports the protective role of human milk against NEC, including through its effects on microbial colonization and mucosal barrier integrity (23–25), but the present data neither confirm nor contradict it. Late initiation of breastfeeding and feeding with infant formula contribute to delayed and abnormal microbial colonization (23). This dysbiotic state compromises the integrity of the intestinal mucosal barrier, increasing its susceptibility to infection; accordingly, breast milk — with its antimicrobial and immunomodulatory properties, including human milk oligosaccharides — has been consistently identified as a protective factor against NEC across multiple studies (24, 25).

No statistically significant association was observed between NEC and in-hospital mortality in this cohort; the crude relative risk of death was 1.52 (95% CI 0.67–3.49). Furthermore, mortality outcomes were missing for 68 of 177 infants (38%), and this missingness was associated with IMV and red blood cell transfusion rather than occurring completely at random, which may have introduced additional bias into this estimate.

The higher Bell stage was associated with increased odds of surgical intervention and mortality in cases of NEC. Markers of systemic compromise were also associated with adverse outcomes, underscoring systemic instability as a key determinant of prognosis in advanced NEC. Recent studies have shown that certain markers can predict progression to surgical NEC, such as C-reactive protein (CRP), which was higher between 24 and 48 h after diagnosis, and platelet counts reaching their nadir within 72 h of NEC diagnosis, with the most pronounced decline observed in the first 24 h (8).

## Conclusions

Taken together, these findings indicate that, in this high-risk neonatal cohort, necrotizing enterocolitis (NEC) showed no statistically significant association with increased in-hospital mortality at the cohort level, an estimate likely underpowered given the small number of events and the substantial proportion of missing outcome data; however, marked heterogeneity was observed within NEC cases.

No association between feeding type and NEC could be established in this cohort. Once NEC had developed, progression to advanced disease stages (Bell IIIB) concentrated the most severe clinical outcomes, including the need for surgical intervention and death, with disease severity and systemic instability emerging as the principal drivers of prognosis. Collectively, this two-level analytical approach identifies markers of clinical severity as the variables associated with NEC occurrence in unadjusted analysis, and disease stage as the principal correlate of clinical outcome among affected newborns.

The risk and protective factors identified in this cohort are consistent with those reported in the literature from high-income countries, and incidence rates are comparable. Rather than reflecting a divergent epidemiology, these findings are compatible with established NEC risk factors also operating in this setting, although the cohort is too small to establish equivalence—a setting now classified as high-income by CEPAL and WHO.

## Strengths

This prospective multicenter cohort study represents one of the few published reports on NEC from a Latin American country, addressing a recognized gap in the regional epidemiological literature.

## Limitations

This study has several limitations. The small number of NEC events (*n* = 14) limited statistical power, and the low events-per-variable ratio (≈1.8) increased the risk of overfitting. Although Firth’s penalized likelihood reduces small-sample bias and addresses separation, it cannot compensate for limited information in the data. Accordingly, adjusted estimates had wide confidence intervals, were sensitive to model specification, and should be interpreted as exploratory and hypothesis-generating. Because we examined several specifications and exposures without correcting for multiple comparisons, individual associations may reflect chance and require confirmation in larger independent cohorts.

Some variables were also difficult to interpret independently. Gestational age and birth weight were strongly correlated; although variance inflation factors were below the conventional threshold of ten, neither variable was independently associated with NEC when both were included together, whereas each was significant when modeled alone. Their coefficients should therefore not be interpreted separately. Similarly, within the NEC subgroup, surgery occurred only in infants with Bell stage IIIB, making disease stage and surgical treatment nearly indistinguishable in this sample and unsuitable as independent predictors of mortality.

Several exposures lacked the temporal precision needed for causal interpretation. Exposure windows were anchored to NEC diagnosis in cases, but no equivalent anchor existed for infants without NEC. Because observation periods differed between groups, we cannot exclude immortal time bias and temporal misclassification. Feeding type was recorded as a single category without timing relative to NEC onset, limiting assessment of reverse causation. We did not collect detailed feeding data, including initiation timing, advancement protocols, and nil-per-os duration, despite their known relevance to NEC risk (26, 27). Transfusion exposure was recorded only as any versus none from birth to NEC diagnosis, without timing relative to onset, limiting evaluation of transfusion-associated NEC within clinically relevant windows such as 48–72 h (28). Future prospective studies should include standardized feeding documentation and time-stamped exposure records.

Mortality outcomes were unavailable for 68 of 177 infants (38%). Because this missingness cannot be assumed ignorable, mortality estimates are restricted to infants with observed outcomes and may be biased in an unknown direction. These estimates should therefore be considered descriptive of that subsample, not the full cohort. Within the NEC subgroup, mortality analyses were based on only four deaths among ten cases with observed outcomes, producing intervals too wide to support quantitative inference.

Finally, residual confounding remains possible, particularly confounding by indication for interventions such as invasive mechanical ventilation and indomethacin. We did not evaluate center as a confounder because, with only fourteen NEC events, adding a center term or stratifying by center would not have yielded interpretable estimates. Differences between units in feeding practices, transfusion thresholds, patent ductus arteriosus management, and other aspects of care therefore remain unmeasured potential sources of confounding.

## Data Availability

The original contributions presented in the study are included in the article/Supplementary Material, further inquiries can be directed to the corresponding author.
